# Outcomes of a novel office-based opioid treatment program in an internal medicine resident continuity practice

**DOI:** 10.1186/s13722-019-0175-z

**Published:** 2019-12-19

**Authors:** Jarratt D. Pytell, Megan E. Buresh, Ryan Graddy

**Affiliations:** 1 Johns Hopkins University School of Medicine, 5200 Eastern Avenue, Mason Lord Building, East Tower, 2nd Floor, Baltimore, MD 21224 USA; 2 Division of Addiction Medicine, Johns Hopkins Bayview Medical Center, 5200 Eastern Avenue, Mason Lord Building, East Tower, 2nd Floor, Baltimore, MD 21224 USA; 3 Department of Epidemiology, Johns Hopkins Bloomberg School of Public Health, Baltimore, MD USA

**Keywords:** Opioid-related disorder, Education, medical, graduate, Office based opioid treatment, Buprenorphine, Primary care

## Abstract

**Background:**

The integration of opioid use disorder (OUD) care and competencies in graduate medical education training is needed. Previous research shows improvements in knowledge, attitudes, and practices after exposure to OUD care. Few studies report outcomes for patients with OUD in resident physician continuity practices.

**Methods:**

A novel internal office-based opioid treatment (OBOT) program was initiated in a resident continuity clinic. Surveys of resident and staff knowledge and attitudes of OBOT were administered at baseline and 4 months. A retrospective chart review of the 15-month OBOT clinic obtained patient characteristics and outcomes.

**Results:**

Twelve patients with OUD were seen in the OBOT clinic. Seven patients (58%) were retained in care at the end of the study period for a range of 9–15 months. Eight patients demonstrated a good clinical response. Surveys of residents and staff at 4 months were unchanged from baseline showing persistent lack of comfort in caring for patients with OUD.

**Conclusions:**

OBOT can be successfully integrated into resident continuity practices with positive patient outcomes. Improvement in resident and staff attitudes toward OBOT were not observed and likely require direct and frequent exposure to OUD care to increase acceptance.

## Background

The prevalence of opioid use disorder (OUD) in the United States is at a record high and continues to rise [1], yet capacity for treatment has not matched demand [2]. Several evidence-based treatment options exist for patients with OUD including treatment with buprenorphine in primary care settings, also known as office-based opioid treatment (OBOT; [3]). The Drug Addiction Treatment act of 2000 (DATA) allows physicians, physician assistants, and nurse practitioners to prescribe buprenorphine for OUD in the outpatient setting to a limited number of patients after completing DATA waiver training [4]. A small proportion of providers have completed the waiver training, and those who have often prescribe below the allowable limits set out in DATA [4, 5].

Common reasons cited by providers for not prescribing buprenorphine include lack of knowledge and experience, and negative attitudes toward patients with OUD [6–8]. Providers with early exposure to treating patients with OUD during training are likely to continue to offer treatment in later practice [9, 10]. However, a recent survey of residency program directors for internal medicine, family medicine, and psychiatry found a minority of programs encourages obtaining a waiver to prescribe buprenorphine for the treatment of OUD [11].

Additionally, there are concerns that office staff may be resistant to integrating treatment for OUD into practice [12–14]. Common concerns are that the patient population will change or that the clinic is not the appropriate place to treat patients with OUD. This is despite evidence showing that patients with OUD (and other substance use disorders) are prevalent in primary care clinics [15, 16].

The widespread integration of substance use disorder care and competencies into graduate medical education is needed [17] and few studies have reported on patient [10, 18–21] or provider outcomes [10, 22–24]. A recent review of graduate medical education models of OUD training found that only 29% of interventions included explicit training in providing medications for OUD (i.e. buprenorphine, methadone, or extended release injectable naltrexone), and a minority of interventions included direct patient care, instead often focusing on didactic learning sessions [25].

The Johns Hopkins Bayview Internal Medicine Residency developed a comprehensive curriculum of OUD competencies including diagnosis, management, and anti-stigma training as part of an effort to incorporate OBOT into the resident physician (referred to as “residents” in this article) continuity practice with the aim to increase patient access and resident exposure to OBOT. In addition, training was provided to clinic staff to improve understanding and acceptance of patients with substance use disorders broadly and OUD in particular. This effort adds to the sparse literature describing educational interventions for OUD training. The primary goal of this study is to describe the structure of the OBOT clinic, provide patient outcomes, and evaluate resident and staff attitudes toward and knowledge of OUD treatment. We hypothesized patient outcomes in the OBOT clinic would be similar to other previously published OBOT programs and resident and staff attitudes and knowledge would move in the direction of increased acceptance and understanding of OUD.

## Methods

### Setting and clinic structure

The Johns Hopkins Bayview Medical Center (JHBMC) General Internal Medicine (GIM) practice has a total of 17 attending providers and is the primary outpatient continuity clinic site for all 48 Internal Medicine residents. There are over 21,000 patient encounters involving 7500 unique patients annually. Prior to March 2017, the practice had not been a site for OBOT and had no faculty preceptors that prescribed buprenorphine for OUD or that completed DATA waiver training.

Residents in the Primary Care/GIM track in the Johns Hopkins Bayview Internal Medicine Residency maintain a second ambulatory practice site. One site, the Comprehensive Care Practice (CCP), is a primary care practice with longstanding experience in OBOT [18]. Three residents have their secondary continuity practice at CCP and complete DATA waiver training as a requirement for practice there. These three residents were chosen as the initial resident providers in the JHBMC GIM Practice OBOT clinic given their experience.

Support for adoption of OBOT in the GIM Practice was provided by the medical director and practice manager, as well as the residency program leadership. The three participating residents alternated monthly coverage for the OBOT clinic. Primary care track residents have 4-week outpatient blocks that start every 12 weeks, so these residents provided continuous coverage without overlapping. Two certified medical assistants and one medical office assistant were assigned to help with the collection of urine drug screens, distribution of prescriptions, scheduling follow-up appointments, and facilitating prior-authorizations. Two practice administrators provided staffing and logistical support.

OBOT visits were initially precepted by DATA-waivered CCP faculty who were not regular preceptors at the GIM clinic; two regular GIM preceptors subsequently completed DATA waiver training during the pilot and this role was transitioned to those faculty. No grants or new funding were used for OBOT, which used the existing practice staff and structure. The CCP preceptors donated their time to precept until regular preceptors took over this role. No additional counseling services or specialized staff were employed during the pilot; all on-site substance use disorder counseling was provided by residents and preceptors. An on-site social worker assisted with behavioral health referrals and connection to community resources. The social worker also helped with vouchers for up to 2 weeks of buprenorphine in situations where a patient’s health insurance required prior authorization for coverage of the medication that would have delayed care; in all cases, patients’ health insurance ultimately covered the cost of buprenorphine. The OBOT clinic operated for 1.5 h per week within a standard 4 h resident session. Time devoted to OBOT was based on anticipated patient volume with consideration of overall ambulatory educational needs of residents to see a diverse array of primary care patients. We report results from the first 15 months of the clinic.

### Resident and staff training

At baseline, all JHBMC internal medicine residents received 4–6 h of training in addressing and treatment substance use disorder through didactics in addition to 4 h of training in motivational interviewing with standardized patients. Specific training on screening and diagnosis of OUD and the use of medications for OUD were emphasized. Curriculum content was rooted in several chief resident experiences with an immersive program to teach addiction medicine [26]. All office staff including certified medical assistants, medical office assistants, and administrators received a one-time one-hour interactive session on the basic pathophysiology, assessment, and treatment of OUD led by a DATA-waivered faculty member prior to the OBOT clinic roll-out.

### Patient Enrollment

Patients receiving primary care through the JHBMC GIM Practice were eligible for referral to the OBOT clinic by their primary care physician. Attending and resident primary care physicians screened patients for OUD, delivered brief intervention, and discussed treatment options including referral to community programs or the internal OBOT clinic. If patients met criteria for OUD and expressed interest in OBOT they would be offered an appointment at the next OBOT clinic session. During the initial OBOT clinic visit, a comprehensive history and physical was completed, urine toxicology screen was obtained, and the prescription drug monitoring program was reviewed. If the resident and attending physician determined a patient to be eligible for buprenorphine, an individualized treatment plan was developed, and the patient was instructed on home buprenorphine initiation; initial clinic follow-up was weekly. Once patients were on a maintenance dose, visit frequency was adjusted based on patients’ response to treatment. Patients remained in care with their previous primary care doctor for all other medical needs.

### Data collection and statistical analysis

All patients who were seen in the OBOT clinic during the study period (March 2017–May 2018) were included in the study. One author (JP) reviewed charts and abstracted de-identified data into an Excel^®^ document located on Johns Hopkins secure servers. The following patient characteristics were obtained: age, gender, race, insurance type, co-morbid conditions, and referral source (attending vs resident). Substance use characteristics included: current alcohol, tobacco, cocaine, stimulant, cannabis, or other hallucinogen use as reported by the patient or discovered on urine toxicology and type of opioid use (prescription opioid, illicit prescription opioid, or illicit opioids such as heroin or fentanyl). Treatment characteristics were obtained: retention (patient was either seen or received a buprenorphine prescription in the last month of the study); induction and maintenance daily dosing (milligram of buprenorphine/naltrexone film dosing equivalents); dosing frequency; and visit intervals.

An outcome measure to determine a good clinical response was adopted from previous literature and described here [18]. The periods after the first prescription were divided into 30-day blocks. Patients were considered in treatment for each block they were prescribed buprenorphine. We used the definition of “inappropriate opioid-positive/buprenorphine negative” if urine toxicology during the block was positive for opioids other than those prescribed, if the patient reported using non-prescribed opioids, or if the urine toxicology was not collected or inappropriately negative for buprenorphine. Otherwise, blocks were “inappropriate opioid negative/buprenorphine positive.” There was no fixed protocol for collecting urine toxicology and clinical decisions regarding toxicology testing were made on a case-by-case basis. The “inappropriate opioid-positive/buprenorphine negative” definition accounts for instances when a urine toxicology was not collected because patients reported return to drug use. Patients who were “inappropriate opioid negative/buprenorphine positive” for more than 50% of the blocks were defined as having a good clinical response. This measure provides information on outcomes of abstinence and reduction in illicit opioid use.

### Resident and staff survey

Two versions of a survey targeted toward residents and clinic staff (certified medical assistants, medical office assistants, nurses, and administrators) were distributed prior to the OBOT clinic initiation (Additional files 1, 2). The surveys were developed by a team of local medical education and addiction-trained faculty and focused on assessment of attitudes toward and knowledge of patients with OUD and their treatment. The same surveys were distributed 4 months after the clinic initiation. This 4-month pre/post survey strategy was selected to capture data within a single cohort of residents given the annual turnover during change of academic year; the project was implemented in March 2017 with surveys distributed in February and June. Surveys were circulated via email to residents and as paper copies to clinic staff.

### Data analysis

Given the small sample size of patients, only descriptive statistics are given for the retrospective chart review portion of the study. Due to missing responses for the follow up survey, paired testing was not feasible. Therefore, we sought to determine if there was a change in the proportion of staff and residents reporting comfort or acceptance on the various measures from baseline to follow up. Likert Scales were dichotomized (1–2 vs 3–5). The Fisher’s Exact test was used to determine if there was a change in the proportion of respondents reporting higher or lower measures over time. This method is preferred when there are few samples in each bin [27]. P values less than 0.05 using a two-sided test were considered statistically significant.

The first buprenorphine prescription was written March 2017 and data was retrospectively reviewed through May 2018, a period of 15 months. This end-date and resulting 15 month period was chosen because of an initial slow referral rate for the first 2 months of the clinic, to coincide with one complete academic year of the clinic, and provide outcomes for patients who were cared for at least 9 months. The retrospective chart review and surveys were independently approved by Johns Hopkins School of Medicine Institutional Review Board.

## Results

### Patient-level data

Table 1 summarizes patient-level data. Over the study period, 12 patients received at least one buprenorphine prescription. The median age was 55.5 years (range 30–78 years). Most patients were male (75%). Seven patients (58%) were retained at the end of the study and engaged in care between 9 and 15 months. Of patients who were not retained, 3 patients transferred to other treatment programs, one patient self-tapered, and one patient was lost to follow-up. A good clinical response was observed in nine patients (75%). Patients were more often referred by residents (75%), reported chronic pain (75%), and had anxiety or depression (33%). Patients reported using prescribed opioids (75%) more often than illicit prescription opioids (25%) or heroin/fentanyl (17%). Three of the patients (25%) reported obtaining illicit buprenorphine. The median buprenorphine initiation dose was 16 mg but ranged from 2–16 mg. For the seven patients who were retained and establish a maintenance dose, the median maintenance dose was 20 mg and ranged from 8–24 mg.

**Table 1:** Patient characteristics and treatment outcomes

N	12
Demographics	
Median age (range)	55.5 (30–78)
Male	8 (75%)
White	12 (100%)
Medicare or private insurance	12 (100%)
Previous opioid used	
Heroin	2 (17%)
Prescribed opioids	9 (75%)
Illicit prescription opioids	3 (25%)
Illicit buprenorphine	3 (25%)
Concurrent substance use	
Tobacco	4 (33%)
Cocaine	5 (42%)
Marijuana	3 (25%)
Retention	
30-day	9 (75%)
90-day	8 (67%)
End of study	7 (58%)
Referral source	
Attending	3 (25%)
Resident	9 (75%)
Dosing characteristics	
Median induction dose (range)	16 mg (2–16)
Median maintenance dose (range)	24 mg (8–24)

### Staff and resident survey data

Survey response rate was 83% for residents (40/48) and 100% for staff (22/22). There were no significant differences for any survey items from pre- to post-survey for residents or staff. Therefore, we have presented results from the pre-surveys only.

Among residents, 90% reported they provided care to at least one patient with OUD and 87% agreed that buprenorphine is an appropriate part of primary care treatment for patients with OUD. Approximately 27% of residents felt comfortable providing outpatient care to patients with OUD. Few residents (30%) believed OBOT is ineffective without formal on-site drug counseling and 7% reported that abstinence from all opioids (including buprenorphine) is the principal goal of treatment for OUD.

Among staff, 47% of respondents agreed that primary care practices are appropriate places to treat OUD; 47% also agreed that the GIM practice was an appropriate place to treat OUD. Most staff (53%) believed OBOT is ineffective without formal on-site drug counseling and 53% reported that abstinence from all opioids (including buprenorphine) is the principal goal of treatment for OUD. Only 41% of staff agreed with the statement that the patient population changes significantly in clinics offering OBOT.

## Discussion

The JHBMC GIM Practice sought to increase the OUD care competencies among clinical staff and residents through educational activities and the integration of OBOT in the resident continuity clinic. We found 58% of patients who received at least one prescription for buprenorphine were retained in the resident OBOT clinic. This is similar to patient outcomes reported at other resident OBOT clinics [10, 18] and across a variety of settings and patient populations [28–34]. We observed a good clinical response in a majority (75%) of patients which was consistent with other experienced OBOT clinical settings [18]. Patients received higher maintenance buprenorphine doses compared to previous studies likely due to the high proportion of OUD developing in the context of chronic opioid therapy for chronic pain [35]. We did not find resident or staff comfort with OBOT improved over time. There were no significant differences in resident and staff knowledge of, or attitudes toward, OUD treatment between surveys at baseline and 4 months after clinic initiation.

This study adds to the limited research evaluating patient and practice-level data from the integration of OBOT into a residency continuity practice. A similarity between the present study and previous reports is the presence of experienced attending physicians to provide oversight and champion project implementation. It is encouraging that although study populations for resident-level OBOT interventions and broader OBOT initiatives are heterogeneous in setting, demographics, and substance use patterns, they consistently show positive outcomes for a vulnerable patient group with OUD.

Our study showed no broad change in resident comfort with providing care for patients with OUD, and across all staff, negative attitudes toward OBOT were prevalent and persistent. This is opposed to a body of literature which demonstrates an improvement of provider knowledge, attitude, and practices after exposure to substance use treatment [24, 36–39]. Our observation is likely related to the limited scope of the intervention—only three residents and three clinic staff members were routinely engaged in care of patients receiving buprenorphine. The small sample of residents did not allow for a comparison between the residents who were and were not routinely engaged in the OBOT clinic. Nonetheless, most residents (87%) believe OBOT is appropriate for primary care. Further expansion of the OBOT clinic has been realized with an increased number of on-site preceptors who have become waivered and residents delivering OBOT; it is possible that the broadened clinical exposure and mentorship will have a greater impact on attitudes and comfort among residents and staff.

This study has limitations which limits its generalizability. First, the sample size was small and consisted of mostly older, white, privately insured patients which reflects the catchment area served by JHBMC. Second, although all residents were responsible for diagnosing OUD and discussing treatment options with their patients, only three were directly responsible for OUD management. Additionally, the total number of residents and staff surveyed was small and the interval between the pre-post surveys was short, which limited resident and staff exposure to the OBOT clinic. As a result of the small sample of residents and short exposure period, it is possible we could not detect differences if they existed between baseline and follow up time points. Finally, this clinic utilized residents with previous OBOT experience to deliver care. It is possible trainees without previous OBOT proficiency may have differences in experience and patient outcomes.

## Conclusion

The resident OBOT clinic described here demonstrates that OBOT can be successfully integrated into resident ambulatory training with positive patient outcomes. Additional studies examining strategies for integration of OBOT into medical training will be crucial in helping to develop a healthcare workforce competent in OUD treatment. As OBOT is expanded into new trainee settings, the impact on residents and staff will be important to measure, and further assessment of characteristics of successful OBOT initiatives will help to provide a scaffold for future efforts to expose learners to treatment models for patients with OUD.

## Supplementary information

**Additional file 1.** Resident physician survey.

**Additional file 2.** Staff survey.

## Data Availability

Requests should be made to the corresponding author.

## Abbreviations

OUD: opioid use disorder
OBOT: office-based opioid treatment
JHBMC: Johns Hopkins Bayview Medical Center
GIM: General Internal Medicine
CCP: Comprehensive Care Practice
