# Supplementary material for: Outcomes of a novel office-based opioid treatment program in an internal medicine resident continuity practice
Source: Addict Sci Clin Pract. 2019 Dec 19;14:46. doi: 10.1186/s13722-019-0175-z (PMC6921403; doi:10.1186/s13722-019-0175-z)
Supplement: Supplementary file 1 — Additional file 1. Resident physician survey. [file 13722_2019_175_MOESM1_ESM.pdf]

Buprenorphine Pilot Resident Assessment

Your completion of this survey or questionnaire will serve as your consent to be in this research study.

1. **What is your current level of training?**
Mark only one oval.

- PGY1
- PGY2
- PGY3
- Other: ______________________

2. **What is your gender?**
Mark only one oval.

- Male
- Female
- Other: ______________________

3. **Residency track**
Mark only one oval.

- Categorical
- GIM

4. **If you are a GIM PGY2 or 3, what is your second site clinic?**
Mark only one oval.

- CCP
- JHCP - White Marsh
- JHCP - Wyman Park/Remington
- JHCP - Dundalk

5. **I have prescribed buprenorphine to patients in a primary care setting.**
Mark only one oval.

- Yes
- No

6. **Opioid use disorder is characterized by the compulsive use of opioids despite adverse consequences from continued use and physical dependence on opioids. I provide primary care to at least one patient with opioid use disorder.**
Mark only one oval.

- Yes
- No
- I don't know

7. **I feel comfortable identifying opioid use disorder in patients I see at RBMG.**
Mark only one oval.

	1	2	3	4	5	
Strongly disagree						Strongly agree

8. **I feel comfortable providing outpatient care to patients with opioid use disorder.**
Mark only one oval.

	1	2	3	4	5	
Strongly disagree						Strongly agree

9. **Buprenorphine (Suboxone) treatment is an appropriate part of primary care practice for patients with opioid use disorder.**
Mark only one oval.

	1	2	3	4	5	
Strongly disagree						Strongly agree

10. **Without on-site formal drug counseling, office-based buprenorphine treatment is ineffective.**
Mark only one oval.

	1	2	3	4	5	
Strongly disagree						Strongly agree

11. **Abstinence from opioids (including buprenorphine) is the principal goal of treatment for opioid use disorder.**
Mark only one oval.

	1	2	3	4	5	
Strongly disagree						Strongly agree

12. **Patients with opioid use disorder who inject heroin should be treated in a more closely monitored setting than patients who use oral prescription opioids.**
Mark only one oval.

	1	2	3	4	5	
Strongly disagree	◯	◯	◯	◯	◯	Strongly agree

13. **Methadone maintenance is a more effective treatment option than buprenorphine for patients with opioid use disorder.**
Mark only one oval.

	1	2	3	4	5	
Strongly disagree	◯	◯	◯	◯	◯	Strongly agree

14. **In practices that start prescribing buprenorphine, the clinic's patient population changes significantly.**
Mark only one oval.

	1	2	3	4	5	
Strongly disagree	◯	◯	◯	◯	◯	Strongly agree

15. **I know enough now to prescribe buprenorphine in the primary care setting.**
Mark only one oval.

	1	2	3	4	5	
Strongly disagree	◯	◯	◯	◯	◯	Strongly agree

16. **I would prescribe buprenorphine to patients with opioid use disorder if I were given proper training and support.**
Mark only one oval.

◯ 1 - Strongly disagree
◯ 2
◯ 3 - Neutral
◯ 4
◯ 5 - Strongly agree
◯ I already prescribe buprenorphine to outpatients with opioid use disorder.

Powered by
Google Forms
