# Supplementary material for: Outcomes of a novel office-based opioid treatment program in an internal medicine resident continuity practice
Source: Addict Sci Clin Pract. 2019 Dec 19;14:46. doi: 10.1186/s13722-019-0175-z (PMC6921403; doi:10.1186/s13722-019-0175-z)
Supplement: Supplementary file 2 — Additional file 2. Staff survey. [file 13722_2019_175_MOESM2_ESM.pdf]

Buprenorphine Pilot Staff Assessment

Your completion of this survey or questionnaire will serve as your consent to be in this research study.

1. **What is your current role in the office?**
Mark only one oval.

- CMA
- MOA
- RN
- Administrator
- Other: ____________________

2. **What is your gender?**
Mark only one oval.

- Male
- Female
- Other: ____________________

3. **Opioid use disorder is characterized by the compulsive use of opioids despite adverse consequences from continued use and physical dependence to opioids. This office provides primary care to patients with opioid use disorder.**
Mark only one oval.

- Yes
- No
- I don't know

4. **Primary care offices are appropriate locations for the treatment of opioid use disorder.**
Mark only one oval.

	1	2	3	4	5	
Strongly disagree	◯	◯	◯	◯	◯	Strongly agree

5. **This primary care office is an appropriate location for the treatment of opioid use disorder.**
Mark only one oval.

	1	2	3	4	5	
Strongly disagree	◯	◯	◯	◯	◯	Strongly agree

6. **Buprenorphine (Suboxone) treatment is an appropriate part of primary care practice for patients with opioid use disorder.**
Mark only one oval.

	1	2	3	4	5	
Strongly disagree	◯	◯	◯	◯	◯	Strongly agree

7. **Without on-site formal drug counseling, office-based buprenorphine treatment is ineffective.**
Mark only one oval.

	1	2	3	4	5	
Strongly disagree	◯	◯	◯	◯	◯	Strongly agree

8. **Abstinence from opioids (including buprenorphine) is the principal goal of treatment for opioid use disorder.**
Mark only one oval.

	1	2	3	4	5	
Strongly disagree	◯	◯	◯	◯	◯	Strongly agree

9. **Patients with opioid use disorder who inject heroin should be treated in a more closely monitored setting than patients who use oral prescription opioids.**
Mark only one oval.

	1	2	3	4	5	
Strongly disagree	◯	◯	◯	◯	◯	Strongly agree

10. **Methadone maintenance is a more effective treatment option than buprenorphine for patients with opioid use disorder.**
Mark only one oval.

	1	2	3	4	5	
Strongly disagree	◯	◯	◯	◯	◯	Strongly agree

11. **In practices that start prescribing buprenorphine, the clinic's patient population changes significantly.**
Mark only one oval.

	1	2	3	4	5	
Strongly disagree	◯	◯	◯	◯	◯	Strongly agree

Powered by

Google Forms
